# Is There a Case for Case-Based Learning in Pharmacology?

**DOI:** 10.7759/cureus.39835

**Published:** 2023-06-01

**Authors:** Thiruganahalli S Padmanabha, Y. D Shilpashree, Ningaiah Ajay, Haradanahalli G Kshamaa, H. L Tejaswi, S. K Raghavendra

**Affiliations:** 1 Pharmacology, Adichunchanagiri Institute of Medical Sciences, Adichunchanagiri University, Balagangadharanatha Nagara, IND; 2 Biochemistry, Adichunchanagiri Institute of Medical Sciences, Adichunchanagiri University, Balagangadharanatha Nagara, IND; 3 Anatomy, Adichunchanagiri Institute of Medical Sciences, Adichunchanagiri University, Balagangadharanatha Nagara, IND; 4 Psychiatry, Kempegowda Institute of Medical Sciences, Bengaluru, IND; 5 Community Medicine, Adichunchanagiri Institute of Medical Sciences, Adichunchanagiri University, Balagangadharanatha Nagara, IND

**Keywords:** case-based learning, post-test, teaching-learning tool, retention, evaluation, intervention, pharmacology, didactic lecture

## Abstract

Background

In a didactic lecture (DL), students listen, take notes, and passively accept the knowledge. Case-based learning (CBL) uses clinical cases for active learning and productive outcome. Although some studies have shown that DL is less effective than CBL, the results were inconclusive. Hence, this study aimed to evaluate the effectiveness of CBL in pharmacology.

Methodology

This study involved 80 second-year medical students divided into two groups. The results of post-test scores and retention test one month later were compared between the groups using multiple-choice questions.

Results

DL showed statistically significant better outcomes in immediate learning compared to CBL in both groups (p = 0.000 and 0.002). Although there were slightly better retention scores for CBL compared to DL in both groups, it was not statistically significant.

Conclusions

DL showed significantly better immediate learning outcomes compared to CBL, with no difference in long-term outcomes for both teaching-learning methods. Hence, DL continues to be the gold standard for teaching pharmacology.

## Introduction

Over decades, teaching medical subjects, especially pharmacology, has been challenging for both facilitators and students during training as it is very volatile. Overall, traditional lecture or didactic lecture (DL) is a commonly used teaching method globally. In the traditional teaching method, students listen, take notes, and passively accept the knowledge, which has been shown to be less effective than other teaching strategies [1]. Students and facilitators noted that traditional teaching methods were not immensely helpful in teaching the subject well and started exploring other teaching-learning (TL) methods [2].

After more than a decade, the Medical Council of India/National Medical Commission implemented competency-based medical education for undergraduate medical students in 2018, emphasizing the need to integrate basic subjects with clinical subjects using case scenarios or case-based teaching. This curriculum also encourages the preclinical and paraclinical disciplines to integrate with relevant clinical disciplines to understand and explore critical thinking skills using case-based discussion and vice-versa [3].

In this context, the goal of case-based learning (CBL) is to prepare and encourage students toward active participation in the learning process for achieving productive outcomes using clinical cases. It links theory to practice through the application of knowledge to cases using inquiry-based learning methods [4,5].

Studies comparing the effectiveness of DL and CBL in knowledge acquisition have yielded inconclusive results, showing no significant differences between the two approaches [6-8]. Therefore, this study aimed to evaluate the effectiveness of CBL over DL in improving learning outcomes, retention levels, and learning satisfaction among second-year medical students.

## Materials and methods

An educational quasi-experimental study was conducted in the Department of Pharmacology after obtaining approval from the Institutional Ethics Committee of Adichunchanagiri Institute of Medical Sciences, B G Nagara (reference number: AIMS/IEC/2259/2019-20).

**Figure 1:**
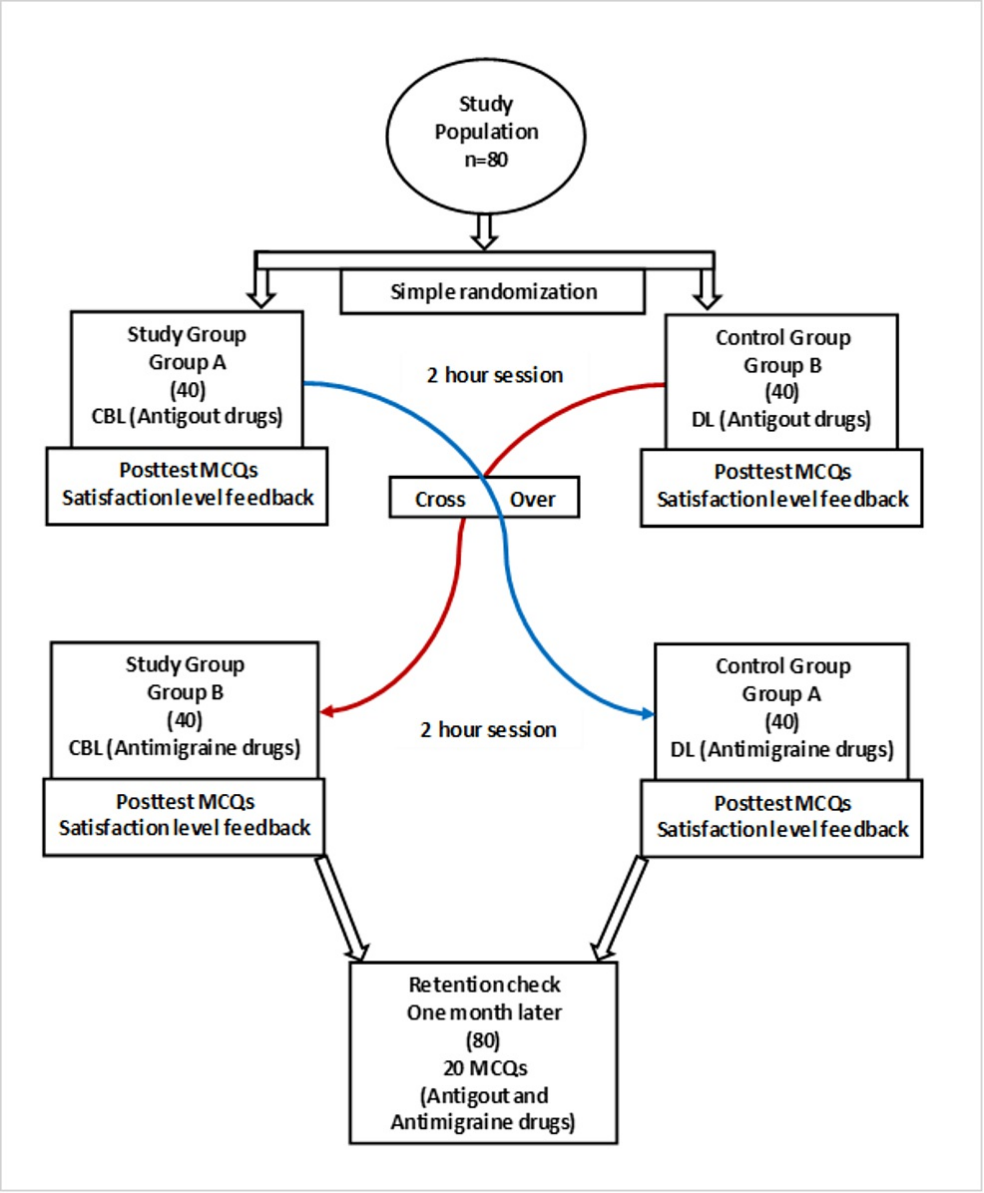
Implementation process of CBL and DL methods. CBL = case-based learning; DL = didactic lecture; MCQ = multiple-choice question

The study included 80 second-year medical students selected using the simple random sampling technique. The research objective and process were explained to the study participants and written informed consent was obtained. The students were randomly allocated into two groups, group A (n = 40) and group B (n = 40), by a random number generator in Microsoft Excel using a 1:1 ratio (Figure 1).

One clinical case vignette, for each session, was prepared by senior faculty, and the content was validated by external subject experts. To assess the outcome of the educational intervention, multiple-choice questions (MCQs) were prepared and validated by the panel of subject experts. Each topic had 10 MCQs, and one mark was awarded for each correct response and zero for each incorrect response. The MCQs were given to all students immediately after the TL session and one month after completing the last intervention. Student satisfaction level was assessed based on a self-designed questionnaire validated by the panel of experts, enquiring whether the DL and CBL methods helped in understanding difficult concepts, encouraging self-study ability, developing problem-solving and reasoning skills, and overall satisfaction with the teaching methods. Each question was scored on a Likert scale of 1 to 5, where 1 denoted very dissatisfied and 5 denoted very satisfied. The average satisfaction scores were considered to compare between the DL and CBL. All sessions were engaged by the senior faculty trained in medical education technology who were sensitized on the effective engagement of students in both DL and CBL methods before the session. The two-week washout period was considered to eliminate the effect of subjective bias among the faculty before the crossover between DL and CBL methods. Figure 1 depicts the implementation process of the study.

Statistical analysis

Compilation and statistical analysis were performed using SPSS version 26.0 software (IBM Corp., Armonk, NY, USA). Continuous data were expressed as mean ± standard deviation (SD). The results between the groups were analyzed using an independent-sample t-test and Mann-Whitney U test. The significance level was set at p-values <0.05.

## Results

**Figure 2:**
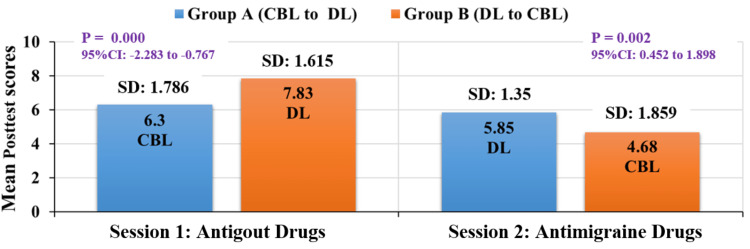
Comparison of mean post-test scores between group A and group B for CBL and DL methods (independent t-test). CI = confidence interval; CBL = case-based learning; DL = didactic lecture

In this study, post-test scores were statistically significant in both group A and group B before and after the crossover, as shown in Table 1, Table 2, and Figure 2.

**Table 1:** Comparison of post-test scores between group A (CBL) and group B (DL) following session one on antigout drugs. No = number of participants; SD = standard deviation; df = degree of freedom; CI = confidence interval; CBL = case-based learning; DL = didactic lecture

Antigout drugs	No	Mean	SD	t-value	df	P-value	95% CI
Group A (CBL)	40	6.3	1.786	-4.005	78	0	-2.283 to -0.767
Group B (DL)	40	7.83	1.615				

**Table 2:** Comparison of post-test scores between group A (DL) and group B (CBL) following session two on antimigraine drugs. No = number of participants; SD = standard deviation; df = degree of freedom; CI = confidence interval; CBL = case-based learning; DL = didactic lecture

Antimigraine drugs	No	Mean	SD	t-value	df	P-value	95% CI
Group A (DL)	40	5.85	1.35	3.234	78	0.002	0.452 to 1.898
Group B (CBL)	40	4.68	1.859				

**Figure 3:**
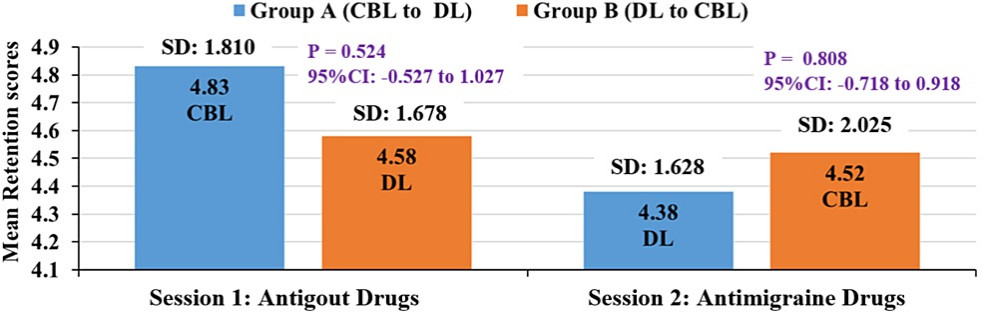
Comparison of mean retention scores between group A and group B for CBL and DL methods (independent t-test). CI = confidence interval; CBL = case-based learning; DL = didactic lecture

Retention scores for group A and group B were slightly higher for CBL sessions, as shown in Table 3, Table 4, and Figure 3.

**Table 3:** Comparison of retention level scores between group A (CBL) and group B (DL) one month after session two on antigout drugs. No = number of participants; SD = standard deviation; df = degree of freedom; CI = confidence interval; CBL = case-based learning; DL = didactic lecture

Antigout drugs - retention level	No	Mean	SD	t-value	df	P-value	95% CI
Group A (CBL)	40	4.83	1.81	0.641	78	0.524	-0.527 to 1.027
Group B (DL)	40	4.58	1.678				

**Table 4:** Comparison of retention level scores between group A (DL) and group B (CBL) one month after session two on antimigraine drugs. No = number of participants; SD = standard deviation; df = degree of freedom; CI = confidence interval; CBL = case-based learning; DL = didactic lecture

Antimigraine drugs - retention level	No	Mean	SD	t-value	df	P-value	95% CI
Group A (DL)	40	4.38	1.628	0.243	78	0.808	-0.718 to 0.918
Group B (CBL)	40	4.52	2.025				

**Figure 4:**
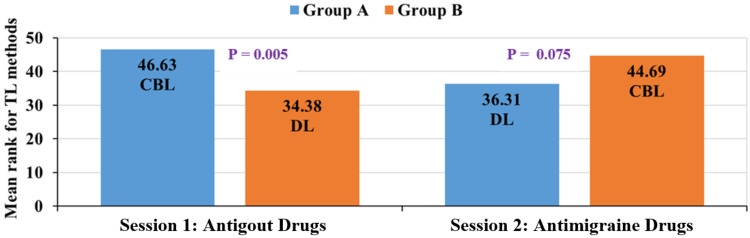
Comparison of perception of satisfaction between group A and group B for CBL and DL methods (Mann-Whitney U test). CBL = case-based learning; DL = didactic lecture; TL = teaching-learning

Figure 4 shows the satisfaction with the TL method. Satisfaction for the group which attended the antigout CBL session was statistically significant (p < 0.05) compared to the group that attended the DL session. Similarly, the group which attended the antimigraine CBL session had higher satisfaction compared to the DL group.

## Discussion

This study was conducted to evaluate the effectiveness of CBL and DL TL methods in teaching pharmacology. Previous studies have compared the outcomes of CBL and DL as effective TL methods and have reported conflicting and inconclusive findings [4-13]. In contrast to the present findings, studies by Cendan et al., Latif et al., Vedi et al., and Sangam et al. concluded that CBL group performance was better than DL [4,5,12,13]. Another study by Chao et al. observed that there was no significant difference between the two TL methods [14]. Therefore, the faculty should not rely on one TL method alone to promote the required educational objectives. Studies by Majeed et al. and Carrero et al. found DL to be the most effective TL method [10,11].

In our study, both groups scored higher for the DL session compared to CBL. Probable reasons that can be derived for their poor outcome in CBL are attributed to their inability to comprehend the clinical case vignette based on previous theoretical knowledge, which requires more time and voluntary active participation, and interaction among peer students and faculty to achieve the learning objective and outcome [15].

Although there were slightly better retention scores for CBL compared to DL in both groups, it was not statistically significant (Figure 3, Tables 3, 4). However, a study done in Turkey dental schools emphasized that CBL improved deeper learning [16]. A study by Ciraj et al. [17] also concluded that CBL helps in long-term retention in teaching microbiology. Hence, they concluded that CBL enables students to acquire, retain, consolidate, and retrieve the concepts in pharmacology by lubricating and linking the neuronal cells in the brain when students are subjected to higher-order mental tasks.

The satisfaction level for TL methods in this study was 46.63% and 44.69% for group A and group B, respectively, who attended CBL, which was higher than that for DL (Figure 4; group A: 36.31% and group B: 34.38%). The CBL method provided a better perception of satisfaction in this study. A study by Kaur et al. [6] also showed that students had a better perception of CBL and there was better attendance in CBL sessions.

Study limitations

This study was conducted on a small sample for a short duration. The outcome might have been influenced by the faculty handling the topic and the topic chosen. Certain confounding factors such as students’ prior knowledge of the topics and their dynamics with the teaching faculty were not assessed and can be considered a limitation of this study. Further, the gap identified in this study can be considered for future evaluation.

## Conclusions

The current interventional study showed that the DL method had significantly better immediate learning outcomes compared to CBL. However, there was no statistically significant difference between CBL and DL in terms of mean retention score. Therefore, DL appears to be the better TL method for teaching pharmacology compared to CBL.
